# The Life Story Book Experience With Residents With Neurocognitive Disorders in Long-Term Care and Their Caregivers: A Mixed-Methods Study

**DOI:** 10.1177/30495334261481199

**Published:** 2026-08-22

**Authors:** Marie-Soleil Hardy, Camille Savoie, Maude Laberge, Chaimaa Fanaki

**Affiliations:** 1 Faculty of Nursing Science, 4440 Université Laval, Québec, QC, Canada; 2 Department of Social and Preventive Medicine, Faculty of Medicine, 4440 Université Laval, Québec, QC, Canada; 3 VITAM, Centre de recherche en santé durable, Université Laval, Québec, QC, Canada; 4 Centre de recherche du CHU de Québec, Axe Santé des populations et pratiques optimales en santé, Université Laval, Québec, QC, Canada

**Keywords:** life story book, neurocognitive disorders, person-centred care, long-term care

## Abstract

**Background:**

Residents with neurocognitive disorders in long-term care facilities experience behavioral and psychological symptoms that affect well-being and relationships. Life story books, personalized compilations of memories, may enhance communication and emotional connection through reminiscence.

**Objectives:**

To examine observed changes in the effects and acceptability of the life story books intervention among residents with neurocognitive disorders and their informal caregivers.

**Methods:**

A convergent mixed-methods design was conducted in three long-term care facilities with sixteen resident–caregiver dyads. Quantitative data were collected before and after a six-month intervention, complemented by qualitative interviews and observations.

**Results:**

Fifteen residents and eleven caregivers completed the study. Quantitative findings revealed changes in positive affect and pleasure, as well as reduced agitated behaviors and an increase in irritability. Qualitative findings highlighted enhanced engagement, communication, and emotional connection. Caregivers reported high acceptability and confidence in using the books, despite occasional emotional ambivalence.

**Conclusion:**

Life Story Book is a feasible and person-centred intervention that shows promise in promoting emotional well-being and meaningful connections in the context of dementia care.

## What This Paper Adds

• Life Story Books can enhance psychosocial outcomes in residents with neurocognitive disorders, improving mood, engagement, and emotional connection.
• Highlights that caregivers find life story books meaningful and manageable, reinforcing person-centred care and caregiver involvement, while emphasizing the need for sensitive facilitation of emotional content.

### Application of Study Findings

• Life Story Books can be implemented in long-term care facilities to support residents’ emotional well-being and reduce agitation or behavioral symptoms.
• Life Story Books offer a practical tool for caregivers and staff to strengthen communication, foster meaningful interactions, and maintain relational continuity despite cognitive decline.

## Background

According to projections from the World Health Organization (World Health Organization, 2022, the proportion of people aged 60 years and older is expected to double by 2050. Similarly, in Québec, older adults are projected to represent 24% of the population by 2031, an increase from 20% in 2021 (Institut de la statistique du Québec, 2021). This demographic shift is leading to a growing demand for residential care, particularly in long-term care facilities (LTCFs). As more people enter LTCFs with higher needs, the challenge is not only clinical care, but creating environments that support the well-being, emotionally, and socially as well.

Residents of LTCFs are older adults with major functional decline and complex health conditions, many living with chronic or degenerative illnesses. Among this population, approximately 70 to 80% are affected by neurocognitive disorders (NCDs), and around 90% of those individuals will experience behavioral and psychological symptoms of dementia (BPSD) during their illness (MSSS, 2018). According to the DSM-5-TR (American Psychiatric Association, 2022), NCDs are characterized by a decline in mental abilities that reduces a person’s capacity to care for themselves independently. There is a decline in performance in one or more cognitive domains such as memory, complex attention, executive functions, language, visuomotor functions, or social skills. BPSD can often be alleviated when underlying causes are properly assessed and when personalized, respectful approaches are applied.

In recent years, long-term care has increasingly recognized that psychosocial needs matter alongside medical needs. One promising approach is reminiscence therapy through the creation of personalized life story books (LSBs). This intervention complements the standardized life history questionnaires completed at admission to collect biographical and medical information and help caregivers understand residents. LSBs go a step further: they are co-created narratives that gather personal stories, photographs, music, videos, and other meaningful memories into a personalized and tangible format. Their purpose is not only to inform but to emotionally resonate, offering a more holistic and human view of the individual (Wills & Day, 2008).

LSB has become a widely recognized component of dementia care, offering a structured yet deeply personal way to stimulate autobiographical memory, preserve identity, and promote social connection (Subramaniam & Woods, 2016). Whether in physical or digital form, these books provide a valuable window into the resident’s care, often giving staff and family an easier way to start conversations and sustain relationships.

A growing body of research, including systematic reviews and empirical studies, supports the positive impact of LSBs for individuals living with dementia (Elfrink et al., 2018; Parker et al., 2020; Subramaniam et al., 2014, 2023; Subramaniam & Woods, 2016). LSBs have been shown to improve communication between residents, caregivers, and family members, as they provide emotionally rich and concrete content to guide conversations (Gridley et al., 2016; Haight et al., 2006). They help personalize care in institutional settings where routines and time pressures can lead to depersonalized interactions. By reinforcing autobiographical memory, LSBs can help residents maintain a sense of continuity and personhood, contributing to emotional well-being while reducing behavioral symptoms (Kindell et al., 2014). In LTCFs, they have been associated with improved quality of life, increased social engagement, and stronger relational bonds (Bohlmeijer et al., 2003).

Ultimately, LSBs, whether on paper or through digital media, offer more than a record of the past. It is a living tool that affirms identity, nurtures emotional connection, and humanizes the care experience. In the face of cognitive decline and institutionalization, these personalized narratives can restore a sense of dignity, agency, and continuity. It also has positive effects on anxiety, depression, negative behaviors, and mood (Subramaniam et al., 2023). By recognizing and honoring the unique life stories of people with dementia, we not only preserve their histories but also affirm their presence, their worth, and their belonging in the here and now.

Despite their promise, LSBs remain underused in institutional settings such as LTCFs. Practical barriers include limited staff time and training, and uneven organizational support. More broadly, innovation implementation in long-term care is consistently complex and highly dependent on local context (e.g., staffing, resourcing, culture, and relationships), which can make it difficult to sustain new practices beyond pilot projects (Windle et al., 2024). Moreover, high-quality longitudinal research demonstrating the cognitive, emotional, and relational outcomes of these interventions in residential care remains limited (Subramaniam & Woods, 2016). While early findings are encouraging, more rigorous evaluation is needed to understand acceptability, feasibility, and clinical effects.

To fully realize the potential of LSB in dementia care within LTCFs, we must move beyond small pilot projects and integrate these tools into everyday practice. LSB should not be viewed as optional or recreational but as a fundamental element of person-centered care. There is a growing emphasis on reaffirming the fundamentals of care, including dignity, respect, privacy, and communication. Within this context, attention is directed toward therapeutic caring and person-centred approaches that place the human experience at the core of care delivery (McCormack & McCance, 2010).

This study examines the acceptability and effects of the LSB intervention among residents in rural northern Quebec LTCFs, contributing to evidence on innovative, personalized psychosocial interventions in dementia care.

## Methods

### Study Design

A convergent mixed-methods design was used to examine the effects of the LSB intervention on residents with NCD in LTCFs and to assess its acceptability among their caregivers. Both quantitative and qualitative data were collected at two time points, analyzed in a complementary manner throughout the study process and subsequently compared to enable data triangulation and corroboration of the findings to provide a comprehensive understanding of the intervention’s implementation and outcomes (Creswell & Plano Clark, 2017).

### Setting

The study was conducted in three LTCFs located in the rural Northern region of Québec. LTCFs involved are public facilities, which means they all operate under the same governance structure, the Integrated Health and Social Services Centre of Côte-Nord (CISSS-CN). Services provided to residents are based on a standardised evaluation of their cognitive and functional skills. Payment is only for the residential portion and is based on the individual’s financial capital so that one’s socio-economic status should not affect the quality of care or the types of services received.

### Participants and Recruitment

The results presented in this manuscript concern two groups of participants: Residents of LTCFs, and their informal caregivers. Residents were eligible if they were aged 65 years or older, had been admitted for less than 6 months, and had a diagnosis of a NCD at stages 2 to 6 on the Reisberg Global Deterioration Scale (Reisberg et al., 1982), indicating mild to moderately severe impairment. Residents with a diagnosis of severe mental illness were excluded. Informal caregivers were defined as relatives or friends who had regular, in-person contact with the resident, while staff participants included employees or volunteers providing care and services in the participating LTCFs. Recruitment was carried out in three sites through the collaboration of the regional administration for elder care at the institution. Resident-caregiver dyads were recruited using non-probability convenience sampling. Recruitment was conducted in collaboration with LTCF managers, who identified residents and caregivers meeting inclusion criteria and were interested in the project. Recruitment continued until data saturation was reached.

Written or verbal informed consent was obtained from caregivers, while resident participation was based on direct consent when possible or proxy consent from a legal representative, complemented by ongoing verbal or behavioral assent during the study.

### Intervention

The LSB consisted of a personalized compilation, prepared either in a traditional paper format (e.g., a photo album) or in a digital format using a photo frame (Pictures 1 et 2). A user guide and short video, developed and validated by the research team with clinical experts, were provided to support participants in co-creating and in using the book. The user guide and video explain, in simple language, which themes to prioritize, how to organize the book, and what information to include to facilitate its use. It also provides questions related to the selected themes and offers strategies for use aimed at increasing the likelihood of positive experiences. Informal caregivers and staff were encouraged to contribute to its creation and to use it in daily care as a reminiscence tool to foster engagement, communication, to reduce BPSD and isolation and to promote the overall well-being of residents. The intervention was implemented over a six-month period.

### Data Collection

Data were collected at two time points: Before the intervention (T1), and at the end of the six-month intervention period (T2). At baseline, sociodemographic characteristics were gathered from all participants. Clinical data including cognitive stage (Reisberg Global Deterioration Scale; Reisberg et al., 1982), and standardized measures of quality of life (QUALIDEM; Ettema et al., 2007) and depressive symptoms (Cornell Scale for Depression in Dementia, short version; Alexopoulos et al., 1988; Barca et al., 2010) were collected at T1 and T2. Participant observation was also carried by trained care staff while using the LSB with participating residents. These observations made it possible to record residents’ levels of engagement and pleasure on a semantic differential scale ranging from 1 to 5 (Bédard et al., 2011). Observers could also provide additional details by describing their observations in greater depth.

Informal caregivers took part in semi-structured interviews at T1 and T2, focusing on their communication and interactions with their loved one. At T2, their perspectives on the intervention’s acceptability were also explored using an interview guide based on the constructs of the Theoretical Framework of Acceptability (Sekhon et al., 2017, 2022) (Table 1). The construct pertaining to intervention coherence was not considered because it did not apply to our intervention.

**Table 1.** Constructs Retained From the Theoretical Framework of Acceptability and Their Definition (Sekhon & Al, 2017)

Construct	Definition
Affective attitude	How an individual feels about the intervention
Burden	The perceived amount of effort that is required to participate in the intervention
Ethicality	The extent to which the intervention has a good fit with an individual’s value system
Opportunity costs	The extent to which benefits, profits, or values must be given up when engaging in the intervention
Perceived effectiveness	The extent to which the intervention is perceived to be likely to achieve its purpose
Self-efficacy	The participant’s confidence that they can perform the behaviour(s) required to participate in the intervention

The interviews were designed to uncover the participants’ current experiences, as well as the challenges and resources that helped them during the creation and use of the LSB.

### Data Analysis

Quantitative data were analyzed using descriptive statistics (means, standard deviations, medians, and proportions) and inferential comparisons between T1 and T2.

Interview transcripts were analyzed using a descriptive interpretive approach (Thorne, 2025) combined with a hybrid deductive–inductive thematic analysis. Initial coding categories were derived from the interview guide and iteratively refined through independent coding of early transcripts by two researchers. The resulting coding framework was then applied to the full dataset. Quotations included in the manuscript were translated for publication.

After the quantitative and qualitative data had been analyzed independently, the findings were integrated to explore convergence, divergence, and complementarity between the two data sources (Creswell & Plano Clark, 2017). Triangulation of quantitative and qualitative findings was performed to enhance internal validity and to provide a nuanced understanding of the LSB intervention’s impact.

### Ethical Considerations

Ethical approval was obtained. All methods were carried out in accordance with relevant guidelines and regulations and Declaration of Helsinki (World Medical Association, 2013). All participants provided verbal or written informed consent to participate in the research project. Prior to recruiting individuals with NCD, consent was first obtained from their legal representatives. Once proxy consent was obtained, care staff and caregivers were required to confirm the resident’s verbal or behavioural assent prior to using the LSB intervention. Confidentiality was maintained by assigning codes to all participants.

## Results

### Sociodemographic Characteristics of Participating Residents

A total of 32 participants (16 residents, 16 informal caregivers) were recruited across the 3 participating LTCFs. At T2, one resident had passed away, resulting in 15 residents completing the follow-up. Eleven informal caregivers participated in the data collection at T2, after four withdrew, two due to unavailability and two due to illness and the death of one resident (Table 2).

**Table 2.** Sociodemographic and Clinical Characteristics of Residents (N = 16)

Characteristics	Mean ± SD or n (%)
**Age**	81.60 ± 8.48
**Gender**	
Male	5 (31.2)
Female	11 (68.8)
**ISO-SMAF score**	10.00 ± 2.37
**Type of neurocognitive disorder**	
Alzheimer’s disease	4 (25.0)
Mixed	1 (6.2)
Vascular	1 (6.2)
Lewy body	2 (12.5)
Other	8 (50.0)
**Other (specific cases)**	
Alzheimer’s & mixed	1 (6.2)
Language Varied Alzheimer/Primary progressive aphasia	1 (6.2)
With BPSD*, not specified	1 (6.2)
Unspecified dementia/NCD	1 (6.2)
Parkinson’s disease	2 (12.5)
Without etiology, NCD with BPSD	1 (6.2)
Diogenes syndrome, mixed NCD with BPSD	1 (6.2)
Sequelae of multiple strokes	1 (6.2)
NCD, unspecified	1 (6.2)

The mean age in the residents’ sample was 81.6 years (range 65–94). Eleven (11) participants were women (68.8%) and 5 were men (31.2%). Alzheimer’s disease was the most common diagnosis (25%), followed by Lewy body dementia (12.5%) and vascular dementia (6.2%). Half of the residents had mixed or unspecified major NCDs. All residents had been diagnosed for more than 3 years.

### Sociodemographic Characteristics of Participating Caregivers

Most informal caregivers were women (68.6%), most often residents’ children (43.8%) or their spouses (37.5%). Their average age was 60.38 years, ranging from 35 to 84 years. Almost all caregivers lived in the same city as the resident, and most reported visiting regularly in person. The majority had post-secondary education (62.5%), and almost half were retired (43.8.3%), while the others were employed either full- or part-time (Table 3).

**Table 3.** Sociodemographic Characteristics of Informal Caregivers (N = 16)

Variables	Mean ± SD or n (%)
**Age**	60.38 ± 15.51
**Gender**	
Male	5 (31.2)
Female	11 (68.6)
**Relationship to resident**	
Son/daughter	9 (56.2)
Grandchild	1 (6.2)
Spouse/partner	6 (37.5)
**Educational level**	
High school diploma or equivalent	8 (50.0)
Apprenticeship/trade school certificate or diploma	1 (6.2)
College (CEGEP) certificate or diploma	4 (25.0)
University degree	2 (12.5)
No certificate, diploma, or degree	1 (6.2)
**Employment status**	
Employed	7 (43.8)
Retired	7 (43.8)
Other	2 (12.5)

### Effects of the Life Story Book on Residents

Quantitative analyses revealed changes in several resident outcomes between baseline (T1) and post-intervention (T2) (Table 4). Given the advanced stage of the residents’ NCD and the absence of control group in this pilot project, these results should be interpreted with caution, as it is difficult to distinguish the specific effects of LSB. Disease stage, assessed with the Reisberg Global Deterioration Scale progressed significantly over the six-month period (p = 0.04), consistent with the natural course of cognitive decline. QUALIDEM scores showed more positive emotional states (p = 0.044) and reduced agitated or tense behaviors (p = 0.021). No significant differences were found for care relationships, negative emotions, social relations, social isolation, or the total score. The Cornell Scale, used to measure depressive symptoms, indicated a significant increase in irritability (p = 0.026), while other items (anxiety, sadness, lack of reaction), and the total score, remained stable. Engagement scores showed no significant change, with many residents maintaining either passive or active engagement, depending on their cognitive status. However, pleasure ratings increased significantly (p = 0.017), and qualitative accounts suggested that the LSB contributed positively by stimulating conversation, evoking memories, and creating moments of joy.

**Table 4.** Effects of the Life Story Book on Residents (N=16 at T1; N=15 at T2)

Measure	T0 Mean ± SD	T2 Mean ± SD	p-value
**Global Deterioration Scale (Reisberg)**	5.50 ± 0.52	5.93 ± 0.59	0.040*
**QUALIDEM**			
– Care relationship	6.31 ± 2.50	5.93 ± 1.98	0.719
– Positive emotions	9.38 ± 2.50	10.40 ± 2.61	0.044*
– Negative emotions	3.69 ± 1.66	3.27 ± 1.87	0.417
– Agitated/tense behavior	4.75 ± 2.72	3.13 ± 2.59	0.021*
– Social relations	6.94 ± 1.81	6.73 ± 1.91	1.000
– Social isolation	6.44 ± 2.10	6.33 ± 2.13	0.855
– Total score	37.69 ± 9.52	35.80 ± 8.43	0.438
**Cornell Scale for Depression in Dementia**			
– Anxiety	0.88 ± 0.72	0.93 ± 0.46	1.000
– Sadness	0.56 ± 0.73	0.53 ± 0.52	0.821
– Lack of reaction	0.38 ± 0.62	0.60 ± 0.51	0.351
– Irritability	0.56 ± 0.73	1.07 ± 0.70	0.026*
– Total score	2.38 ± 2.00	3.13 ± 1.51	0.246
**Engagement**	3.63 ± 1.09	3.60 ± 0.83	0.821
**Pleasure**	3.19 ± 0.91	3.83 ± 0.92	0.017*

*Statistically significant at p < 0.05.

The qualitative results revealed effects on residents’ anxiety and agitated behavior. Despite residents’ NCD, observations reported by care staff during LSB use show that residents responded positively when LSB was used as an intervention strategy.

“Before bedtime, it relaxes him, and that's when I get to spend some time with him and show him his family and the book.” (S1A.R1)

Several residents showed signs of pleasure and engagement when presented with their book, particularly when looking at photographs of family members or significant life events:

*“*The book helped to continue stimulating him. When we show him the photos, the big smile, you can see it makes him feel good.” (S2B.P2)

For some, the LSB served as a starting point for conversations, which could then expand into broader exchanges. It was often described as easy to use and not burdensome. One caregiver explained how the book facilitated interaction:

“Of course the photographs are effective… she will tell us details about her life story, she will really talk about her wedding, about her children.*”* (S1A.R2)

Caregivers also highlighted that the LSB reassured them by offering a structured way to interact, especially during visits when conversation was otherwise difficult.

“You have to feed the conversation… even if he responds negatively, you continue.*”* (S2A.R3)

### Acceptability of Intervention Through Caregivers’ Perspectives

At the end of the intervention, caregivers (N = 11) were interviewed regarding the LSB’s acceptability. Quantitative ratings on a 0–10 Likert scale were complemented with qualitative comments. Table 5 summarizes the mean ratings across key dimensions.

**Table 5.** Caregivers’ Acceptability Ratings of the LSB (N = 11)

Dimension	Mean ± SD
**Attitude**	8.73 ± 1.85
**Ethicality**	8.73 ± 1.54
**Burden**	
Development of the LSB	4.77 ± 2.77
Use of the LSB	1.45 ± 2.43
**Opportunity costs**	3.82 ± 1.19
**Perceived effectiveness**	
Improved communication/interactions	5.18 ± 3.89
Improved quality of care	5.00 ± 3.17
**Self-efficacy**	
Self efficacy to develop the LSB	8.09 ± 1.81
Self efficacy to use the LSB	8.55 ± 2.02

#### Attitude

This theme reflects caregivers’ appreciation of their participation in the experiment. On average, they were very positive about their experience (8.73 ± 1.85).

In general, caregivers highly appreciated creating and using the LSB with their loved one, as it provided happy moments with the resident and prompted them to reminisce and share more conversations:

“I also liked preparing it with my mother because we exchanged on things we had never talked about before. That’s what I would say.” (S2A.P3)

However, some were initially reluctant to participate. Reported reasons included lack of time or mental load, as the creation of the book was perceived as an added task in their demanding daily lives:

“At first I found it… well, it was time that was missing, but once I got into it, it went well.” (S2B.P1)

Several factors encouraged caregivers to overcome this reluctance, such as seeing an inspiring example of a LSB created by someone they knew or the desire to please their loved one:

“The first time they talked to me about the project, I wasn’t interested. Later, I saw the book one of my friends made for her husband and thought it was a very good and beautiful idea.” (S1A.P7)

However, one caregiver found the creation of the LSB emotionally difficult, given her father’s neurodegenerative illness and the fact that her mother had died the previous year:

“Because in my case, I lost my mother, and I lost my father in another way… you understand? So, when I dove into the photos, it was really hard.” (S1A.P4)

Despite this, she emphasized that even limited reactions from her father to the book were like small rewards that she cherished:

“It’s a beautiful memory of my dad in the nursing home, when he opened it and reacted… a beautiful memory in my daughter’s heart.” (S1A.P4)

#### Ethicality

Caregivers rated the alignment of the intervention with their personal values very highly (mean = 8.73 ± 1.54). Overall, caregivers considered that the LSB was strongly consistent with their personal and family values. Several noted that photos were important in their families or for the resident, and that the LSB represented family, memories, and the resident’s life.

“Yes, it’s part of our values, because X loved looking at photos…” (S1A.P1)

For one caregiver, whose father loved reading, the paper format of the book was particularly meaningful:

“Reading was always important. I didn’t want a digital book, I wanted a paper book, because even though my dad didn’t have much formal education, he read newspapers every day and big books, encyclopedias.” (S2A.P3)

#### Burden

##### Perceived Efforts to the Development of the LSB

Caregivers rated the effort required to create the book as moderate (mean = 4.77 ± 2.77). Several emphasized that it was more a matter of finding time than difficulty in the process, such as the search for photos:

“I wouldn’t quantify it as effort, more as taking the time. It wasn’t hard, but it took time to build. Collecting photos, framing them, placing them, it’s just time.” (S2B.P4)

For some, the main burden was emotional, particularly when dealing with loss:

“A lot of emotional effort. Not so much time and energy, it was the emotions when looking at wedding photos of my parents, or when planning what to include.” (S1A.P4)

Family dynamics also added a layer of responsibility:

“I felt a small obligation to consider everyone who would see the album… if I included old photos of building the house, I had to think of uncles, aunts, siblings.” (S1A.P4)

##### Perceived Efforts to Use the LSB

The effort to use the book was rated very low (mean = 1.45 ± 2.43). Most reported no effort at all, describing it as simple and enjoyable:

“It didn’t require effort, sometimes I looked at it with him, zero! Just take out the album and talk about the photos. It was pleasant, not difficult.” (S1A.P1)

#### Opportunity Costs

Overall, both financial and time costs were perceived as limited, generally low and worthwhile. Caregivers rated perceived costs as low (mean = 3.82 ± 1.19). Most reported little or no financial expense, since materials were provided, though some covered printing costs:

“Zero money. I did it on the computer, scanned photos, made a montage, no extra costs.” (S1A.P4)

“It was the photo printing. Maybe under $10.” (S2B.P2)

In terms of time invested in creating the book, it varied widely: Some completed it in less than 10 hours, others spent more than 20 hours, often due to photo sorting.

“I did it over three weekends, including photo searches. It wasn’t just making it, but reflecting on it. Sometimes I had to stop, cry, or take a break.” (S1A.P4)

Time spent using the book also varied. Some families used it daily or multiple times per week, while others only occasionally. Duration of use depended on the resident’s attention span and state:

“We use it almost daily, sometimes twice a day depending on his energy.” (S2A.P2)

“Ten, fifteen minutes, when I see his attention drifting, I stop.” (S2B.P1)

Importantly, they considered it feasible and not an additional burden, as it fit into their regular interactions with their relative.

#### Perceived Effectiveness

Caregivers gave a moderate score for interaction and communication quality (mean = 5.18 ± 3.89). Some observed strong positive effects:

“When I show her the photos, she smiles and communicates more. She adds sentences she wouldn’t have said otherwise.” (S2B.P2)

Others saw little reaction, especially due to some residents advanced NCDs condition:

“I can’t say I saw much reaction, not really.” (S1A.P1)

Some reactions were emotional, including sadness or tears:

“At the beginning, she cried and said she wanted to go home… but afterward it was mostly positive.” (S2B.P2)

The caregivers’ perception of care quality improvement was also rated moderate (mean = 5.00 ± 3.17). Some reported that staff used the book to calm residents or personalize care:

“One time he wasn’t soothed, and they managed to bring him back by showing him the book.” (S1A.P4)

Others noted little effect, often because they reported that staff rarely used the book:

“If staff used it, it could improve care by giving them conversation topics, but they didn’t.” (S2B.P4)

Thus, while some perceived benefits for care, effectiveness depended on staff engagement.

#### Self-Efficacy

Caregivers rated their confidence as high in the LSB development process (mean = 8.09 ± 1.81). Some were initially uncertain but gained confidence, while others felt confident from the start, due to previous experience with scrapbooking or photo albums:

“I thought I was bad at crafts, but as I worked on it, I gained confidence and had fun.” (S1A.P6)

Confidence in its use was very high (mean = 8.55 ± 2.02). Nearly all caregivers felt competent, proud, and happy to use it:

“I was proud of myself, when you see it works, it’s rewarding.” (S2B.P1)

Only one caregiver reported difficulties, citing her father’s limited attention and her own emotional challenges:

“It was hard to keep his attention on the pages, and hard not to cry looking at the photos.” (S1A.P4)

## Discussion

This study set out to examine the effects of the LSB intervention on residents living with NCDs in LTCF and the acceptability of the intervention by their caregivers. The findings suggest that the LSB intervention may offer psychosocial benefits for residents with dementia, including better mood outcomes, improved communication, and strengthened relationships with staff and family, thereby supporting quality of life in long-term care. Care staff and informal caregivers commonly report LSBs as meaningful and feasible tools that help them better understand the person behind the diagnosis and tailor person-centred care. When implemented with attention to consent, ownership, and sensitive content, LSB approaches align with ethical person-centred practice. Overall, these findings are consistent with, and extend, the broader evidence base on reminiscence-based interventions in dementia care (Elfrink et al., 2018; McKeown et al., 2010;Subramaniam et al., 2014; Woods et al., 2018).

Quantitative findings demonstrated changes in psychosocial indicators measured by the QUALIDEM, including positive affect and pleasure in daily interactions, with a noticeable reduction in agitated behaviors. These results align with evidence from systematic reviews showing that reminiscence therapy can improve mood, affect, and quality of life in dementia (Subramaniam & Woods, 2016; Woods et al., 2018). Qualitative accounts reinforced this trend, as staff and caregivers observed concrete signs of engagement and pleasure through smiles, vivid eye contact, and spontaneous narratives. Such responses illustrate the potential of the LSB to stimulate preserved autobiographical memory and create moments of meaningful connection, even in institutional contexts that often risk depersonalization. It is also important to acknowledge that the observed improvements in positive affect and engagement may not be attributable exclusively to the LSB intervention, as they may partly reflect increased attention with residents, a phenomenon commonly referred to as the Hawthorne-type effect (Sedgwick & Greenwood, 2015).

At the same time, informal caregivers noted that residents’ responses varied by dementia stage: those in earlier stages more often recognized photos and engaged, while those in advanced stages showed few reactions. This heterogeneity is consistent with prior research linking responsiveness to cognitive and communicative capacity (Kindell et al., 2014). Relatives’ emotionally shaped expectations, often focused on memory recall, may have limited their perception of interaction quality. Although depressive symptoms increased over time, this may be attributed to the natural progression of NCDs rather than the intervention, underscoring the need to interpret outcomes within the context of disease evolution. This is consistent with evidence that depressive symptoms can fluctuate or worsen as NCDs progress, reflecting both neurobiological changes and increasing functional and psychosocial burden associated with disease advancement (American Psychiatric Association, 2022). This also highlights the importance of using control or comparison conditions in future studies to better isolate intervention effects from disease-related changes over time.

For residents with advanced dementia and severe language deficits, the LSB may need to be complemented by other reminiscence tools, such as music, video, or multisensory stimulation. Music has been shown to evoke rapid, emotionally rich memories in Alzheimer’s disease (El Haj, Fasotti, & Allain, 2012El Haj et al., 2012), suggesting that a multimodal toolkit may extend the benefits of life story work to individuals who are less able to engage with text or photographs.

Caregivers generally reported a favorable acceptability of the LSB, with particularly strong ratings for attitude, ethical alignment, and self-efficacy. Beyond the quantitative data, they emphasized that creating the book allowed them to reclaim a meaningful role in their relative’s care, often disrupted by the transition to institutionalization. Contributing to the book provided them with a sense of active participation, helping some to better accept the placement of their loved one while maintaining continuity with their past. This finding resonates with literature showing that reminiscence work not only benefits individuals with dementia but also strengthens family identity and caregiver resilience (Bohlmeijer et al., 2003; Haight et al., 2006).

Nevertheless, caregivers also described ambivalent experiences. For some, revisiting family history was painful, emphasizing the contrast between the resident’s past vitality and present decline. This tension illustrates that reminiscence can evoke both comfort and grief, depending on the memories engaged. Prior studies have similarly cautioned that life story work may trigger sadness or loss if not carefully facilitated (Haight et al., 2006). These accounts suggest that structured guidance could help families navigate emotional ambivalence, ensuring that the intervention remains supportive rather than overwhelming.

Perceptions of effectiveness were moderate, particularly regarding improvements in communication and care quality. While some caregivers observed better conversations, more smiles, and recognition, others reported little change. This variability likely reflects both disease progression and facilitation quality. The LSB appears to function best as a relational tool. Its impact derives not from the book itself but from the interactions it stimulates when used actively with residents. This hypothesis aligns with the broader literature suggesting that the benefits of reminiscence depend heavily on social context and facilitation (Subramaniam & Woods, 2016). Some caregivers reported little effect, often because they noted that staff rarely used the book. These findings are consistent with previous studies showing that the successful implementation of interventions focused on reminiscence and life stories depends on their integration into the natural rhythm of daily care (Doran et al., 2019).

Finally, caregivers reported high self-efficacy in developing and using the LSB, overcoming initial doubts about their abilities, which supports its potential for wider dissemination. Minimal training requirements may aid sustainability. Beyond resident benefits, the rewarding experiences described may also enhance caregiver resilience. Future research should examine whether life story work improves caregiver mental health, reduces burden, and supports more positive interactions.

## Strengths and Limitations of the Study

This pilot study has strengths and limitations that should inform interpretation of its findings.

Key limitations include the absence of a control group, which limits causal attribution of the observed effects. Additional constraints include recruitment challenges due to the vast geography, which reduced the diversity of institutional contexts. Sample sizes were modest, appropriate for exploratory work but limiting generalizability. Residents also varied widely in dementia severity, affecting consistency of engagement. In addition, the short follow-up period prevents conclusions about long-term sustainability, and the absence of a formal control group limits causal attribution.

**Figure 1.**
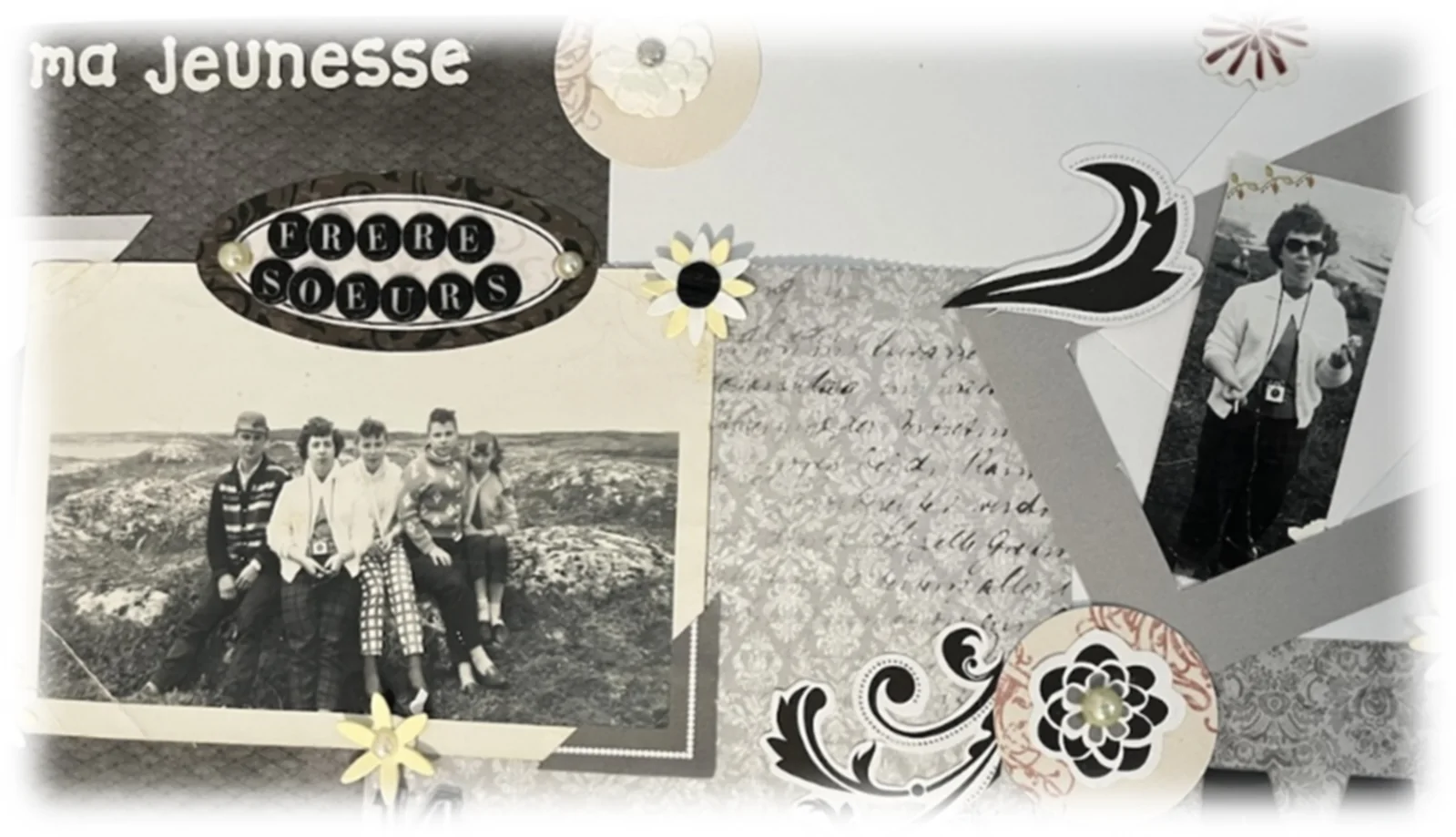
Images from Ludivine Guillemette’s photo album, featured in a Radio-Canada report by Chicoine McKenzie (2025) and published with the permission of Sylvie Collard

**Figure 2.**
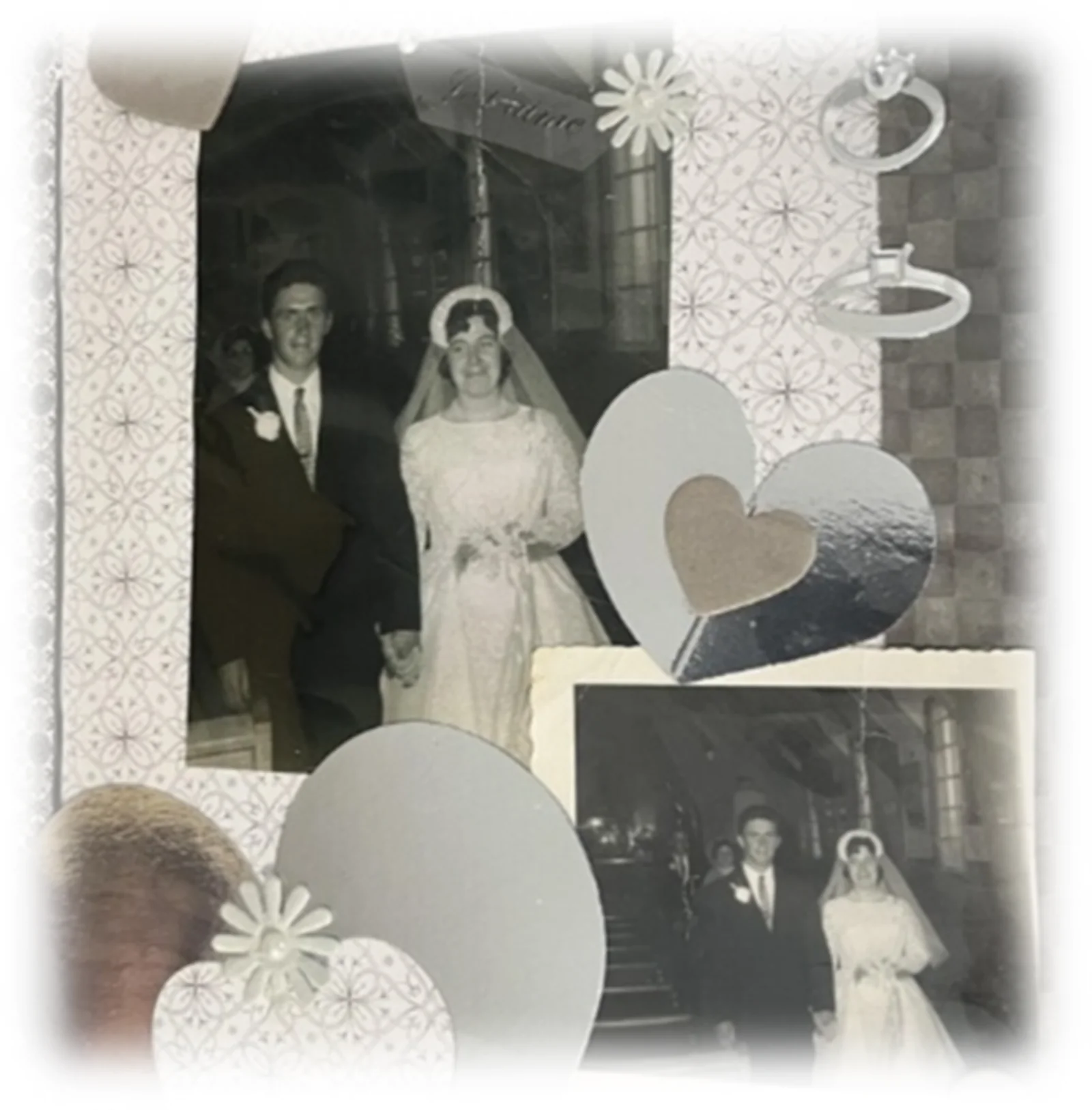
Chicoine McKenzie, R. (2025-05-07). Adoucir la vie en CHSLD grâce à un album photo. Radio-Canada. https://ici.radio-canada.ca/nouvelle/2163508/aine-personne-age-chsld-vieux?

Despite these constraints, the study has key strengths. Its mixed-methods design combined quantitative measures with rich qualitative accounts, enhancing credibility through triangulation. Including residents across cognitive stages, though complex analytically, reflected real-world long-term care. Caregivers found the intervention highly acceptable, reporting increased role value and more meaningful interactions, supporting its feasibility (Figures 1 and 2).

## Conclusion

This study shows that the LSB is a feasible, acceptable person-centred intervention in LTCFs that shows promise for supporting positive affect and pleasure and reduced agitation in residents with NCDs. By encouraging the sharing of personal memories, experiences, and identities, it may help facilitate more positive interactions between residents and informal caregivers, while contributing to a more individualized and compassionate care environment. Caregivers valued its emotional and relational benefits, though effects varied by dementia severity. Despite methodological limitations, findings suggest the LSB’s potential to enhance well-being and connection. Further controlled studies are needed to confirm outcomes, better understand the mechanisms underlying observed changes and support the sustainable integration into daily practice across diverse care settings. As a simple, low-cost tool, the LSB helps preserve personhood and humanize dementia care.

## Data Availability

The datasets generated and/or analyzed during the current study are not publicly available due to ethical concerns and restrictions imposed by the ethics committee, and prior agreements with participants, but are available from the corresponding author on reasonable request.*
